# Incidence of Bacterial Chondronecrosis with Osteomyelitis Lameness in Commercial Duck Flocks in Mojokerto, Indonesia

**DOI:** 10.3390/ani15243632

**Published:** 2025-12-17

**Authors:** Andi Asnayanti, Siti Azizah, Anif Mukaromah Wati, Ahmad Ridwan, Ahmad Arman Dahlan, Dinda Rosalita Asmara, Anh Dang Trieu Do, Adnan Alrubaye

**Affiliations:** 1 Center of Excellence for Poultry Science, University of Arkansas, Fayetteville, AR 72701, USA; aasnayan@uark.edu (A.A.); ad086@uark.edu (A.D.T.D.); 2 Animal Science Faculty, Brawijaya University, Malang 65145, Jawa Timur, Indonesia; siti.azizah@ub.ac.id (S.A.); anifwati@ub.ac.id (A.M.W.); ahmadridwan.ea@gmail.com (A.R.); 3 Agricultural Department, Mojokerto 61361, Jawa Timur, Indonesia; areksmojos@gmail.com (A.A.D.); dindarosalita1@gmail.com (D.R.A.); 4 Cell and Molecular Biology Program, University of Arkansas, Fayetteville, AR 72701, USA

**Keywords:** Chondronecrosis, osteomyelitis, lameness, ducks, bones

## Abstract

Bacterial Chondronecrosis with Osteomyelitis (BCO) lameness is an infection of opportunistic bacteria in the structural skeletal bones impacting multiple animal species, particularly poultry species. BCO lameness results in significant financial losses to industrial poultry production and increases the risk of foodborne illnesses, posing a major threat to consumers’ food safety. As BCO lameness is an inherent risk of fast body weight gain in poultry species, especially broiler chickens, abundant studies have been conducted in broilers and turkeys. Nevertheless, BCO lameness incidence in ducks remains elusive. Thus, this is the first survey investigating the prevalence of BCO lameness cases in ducks. The survey was conducted in commercial duck farms in Indonesia, the fourth biggest duck-producing country globally. Two hundred birds from four commercial duck farms in Mojokerto, East Java, Indonesia, were necropsied to examine their lameness lesions in the femoral head and proximal tibia. Of the 44% birds showing BCO lameness lesions, 3% were evidently clinically lame birds, particularly exhibiting limping gait. Femoral head separation (FHS) and tibial head necrosis (THN) are the most frequently observed lesions in ducks, representing a mild-to-moderate BCO lameness state. Based on the results of this study, intervention measures to boost the immune system and skeletal bone integrity of ducks are urgently required.

## Simple Summary

Duck meat is one of the key protein resources in the poultry industry. Approximately 80% of the global duck supply is dominated by the Asian market, including Indonesia. Musculoskeletal disorders are commonly encountered in birds across taxa, including ducks, and constitute a substantial threat to animal well-being and meat quality. Generally, ducks are smaller than broiler chickens. Considering the high rate of Bacterial Chondronecrosis with Osteomyelitis (BCO) lameness in commercial broiler farms, it is questioned whether the prevalence of osteomyelitis-associated lameness in ducks is also high. Thus, this study aims to survey the rate of BCO lameness incidence in the commercial duck flocks in Mojokerto, Indonesia, which may potentially lay the foundation of future investigation into this concern.

## 1. Introduction

Since 1961, the trend of global meat consumption has gradually shifted from beef to poultry meat [1,2]. By 2030, the demand for poultry meat per capita is estimated to rise by 271% in South Asia, 116% in Europe and Central Asia, 97% in the Middle East and North Africa, and 91% in East Asia and the Pacific, compared to the poultry meat consumption rate in 2020 [3]. By 2034, 55% of the global meat supply will be dominated by Asia, accounting for a 15 Mt increase in poultry production [4].

Chickens, turkeys, ducks, geese, and quail are the main sources of global poultry protein. In Asia, duck meat constitutes 10% of the total poultry meat production [5]. The largest duck populations are found in China, Vietnam, Bangladesh, and Indonesia [6,7], with the production of duck meat in Indonesia reaching 59 million in 2024 [7]. A significant increase in duck meat production in the past 10 years can be attributed to the spread of information on culinary tourism and lifestyle through social media [8,9].

Duck meat is high in nutrients, including essential amino acids, high polyunsaturated fatty acids, and a balanced ratio of omega-6 and omega-3 [10,11]. Compared to other poultry species, ducks are more resilient due to their superior immunity against diseases [12] and are easy to mate and manage in a colony, particularly in wet areas [12]. Manila duck, Khaki Campbell, Peking duck, and their crossbreed offspring have promising potential in Asian markets [13]. Mojokerto is one of the central duck production areas in East Java, Indonesia, where the domestic duck population is estimated at more than 5 million [14]. Mojosari (*Anas javanica*) and Alabio (*Anas platyrhynchos Borneo*) are indigenous breeds in Mojokerto, possessing some advantageous traits such as heat stress resistance and adaptability to tropical environments, but they are small in size [15] compared to Peking ducks from China, which grow to large sizes quickly [16]. The female Mojosari duck has been proven to reach an average body weight of 1.58 ± 0.09 kg, whereas the female Alabio duck has an average body weight of 1.25 ± 0.12 kg [15]. As such, duck farmers in Indonesia have developed hybrid duck breeding, crossing two or more different types of ducks, to select for advantageous traits. For example, farmers in Mojokerto cultivate MP (Mojosari–Peking) hybrid ducks from a cross of female Mojosari ducks and male Peking ducks [17].

Despite these efforts, several factors may impede the ducks from reaching their normal body weight, including inadequate nutrient intake, inappropriate rearing or housing system, and disease outbreaks [18,19]. Generally, farmers practice three types of duck-rearing systems. First is an intensive rearing system, where ducks are highly confined in controlled cages and supplied with a diet rich in nutrients. Second is a semi-intensive rearing system, where ducks are confined but are still allowed to graze, especially after a paddy harvest. Third is an extensive or traditional rearing system in which ducks have unlimited access to outdoor areas, usually in harvested rice fields, to feed themselves. In the latter rearing method, the ducks are allowed to forage, dust bathe, and express their natural social behaviors [20,21,22,23].

In addition to the ecological influence, infectious diseases from viral, bacterial, or parasite infection also highly affect duck performance. Musculoskeletal diseases also commonly occur in ducks, with substantial impact on animal welfare and the quality of poultry meat. Pododermatitis, osteoarthritis, and nutritional deficiencies are musculoskeletal disorders causing lameness in poultry flocks [24,25]. Another leg condition affecting poultry is osteomyelitis, an inflammatory disease in the skeletal system due to pathogen infections, leading to progressive bone necrosis, which is generally known as Bacterial Chondronecrosis with Osteomyelitis (BCO) lameness [26,27,28,29]. Health issues associated with musculoskeletal bone impose a substantial problem in duck meat due to a loss of normal weight gain [30]. Infections of opportunistic microorganisms in the structural skeletal bone, causing BCO lameness, can be fatal and lead to morbidity because the skeleton is the largest system in the body [26,29]. Unlike BCO lameness in broilers, which has been intensively investigated, hardly any studies have been conducted on the osteomyelitis-associated lameness in ducks. Thus, this study aims to survey the rate of BCO lameness incidence in the duck population in Mojokerto, Indonesia, as one of the first significant efforts in BCO field research, which may potentially lay the foundational groundwork for future studies focusing on this aspect.

## 2. Materials and Methods

### 2.1. Animal Use Statement

**Figure 1:**
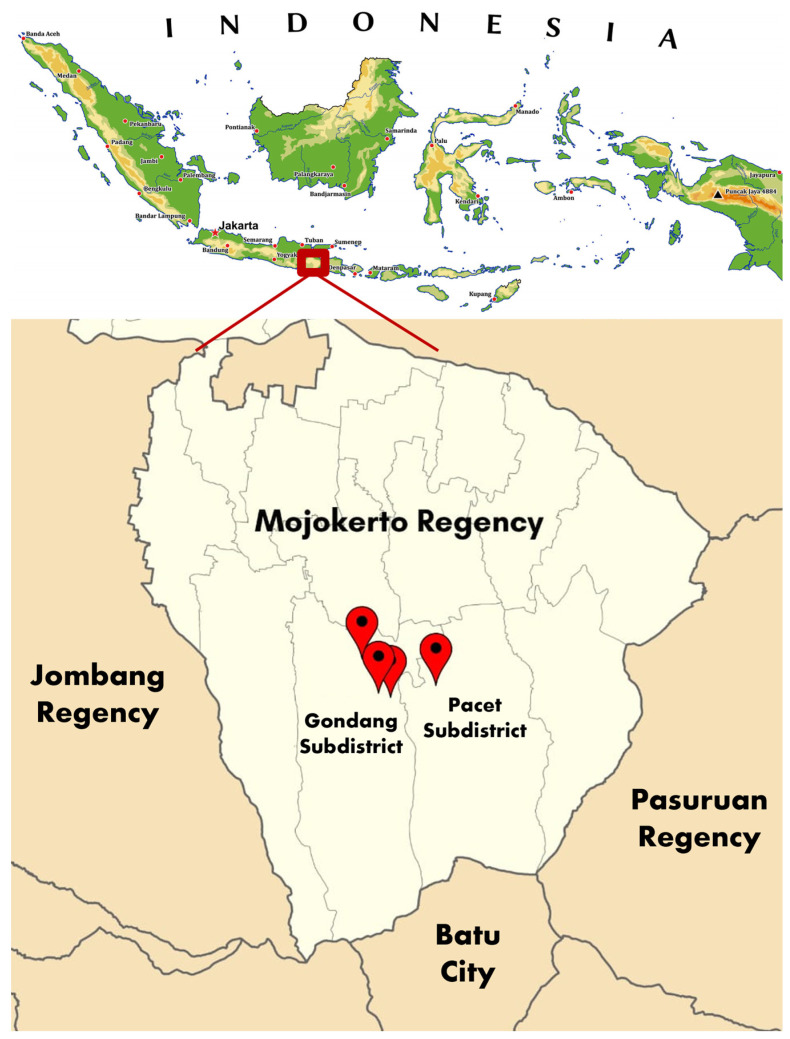
Map of four survey sites of BCO lameness in commercial duck farms in Mojokerto, East Java, Indonesia. The red dots indicate four locations of the survey sites.

A survey of BCO lameness incidence was conducted in commercial duck farms in Mojokerto, East Java, Indonesia, in September 2025. Figure 1 presents the map of the survey sites in Mojokerto. The Ethical Clearance for this survey was examined and issued by the Malang State University, with decree No.19.06.17/UN32.14.2.8/LT/2026.

### 2.2. Survey Design

**Figure 2:**
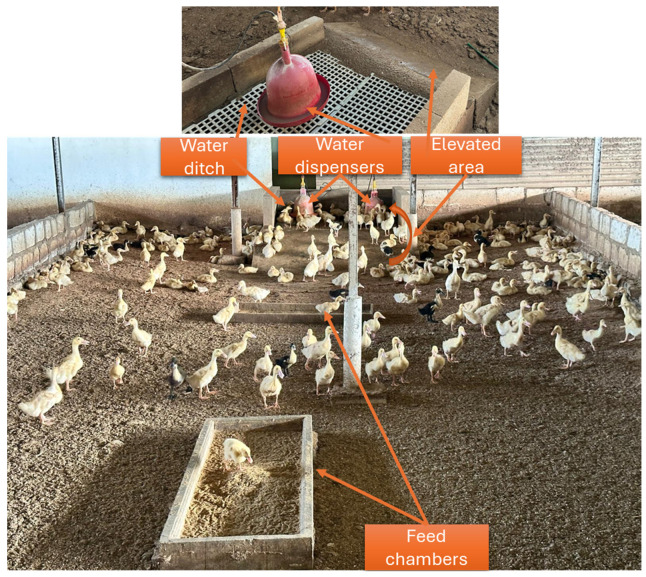
Layout of the barn sized 11 × 15 m^2^ for 250 birds, with water dispensers in the hilly area, a water ditch underneath, and feeding chambers in the opposite areas.

The survey was carried out on four farms growing MP hybrid duck breeders. The ducks were reared in a semi-intensive housing system with a density of 1.51 m^2/^bird at the age of 35–42 days. The production houses used wood shaving litter floors, and the litter floors were changed every 5–6 months, at the end of 3 production cycles. Ducks had ad libitum access to feed and water. The picture of the water dispensers and feed chambers layout is in Figure 2. The water dispenser was located in a hilly site with a water ditch underneath to prevent flooded litter from spillage. The ducks were fed with a commercial New Hope 831 Green Footprint diet (PT. New Hope Jawa Timur (Mojokerto), Indonesia) from d0 to d10 in the scramble form. Starting from d11 until slaughtering age, the ducks were fed a diet of rice mill byproducts mixed with commercial HI-PRO-VITE144 feed concentrate (Charoen Pokphand Indonesia Tbk, Sidoarjo, Indonesia) at a 5:1 ratio. The slaughter age of ducks in East Java is 40–42 days of age.

**Figure 3:**
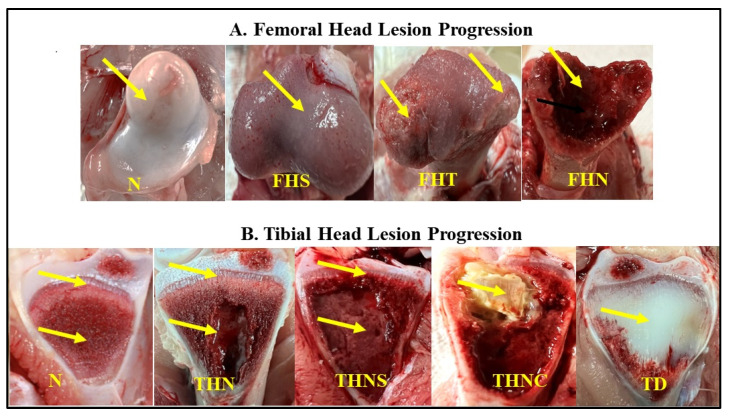
Categorization of femoral (**A**) and tibial (**B**) lesion progressions. Yellow arrows represent the differences in the lesion severity in each category. N = Femur head and proximal tibia appear entirely normal; FHS = Proximal femoral head separation (epiphyseolysis); FHT = Proximal femoral head transitional degeneration; FHN = Proximal femoral head necrosis; THN = Proximal tibial head necrosis; THNC = Proximal tibial head necrosis caseous; THNS = Proximal tibial head necrosis severe TD = Tibial dyschondroplasia.

The sample size was 200 ducks aged 35 to 37 post-hatching days from four commercial farms (50 birds/farm). Specifically, three farms had birds at 35 days of age, and one farm had birds at 37 days of age. The birds were randomly taken, and their body weights were measured in groups of 10 birds. Then, they were necropsied one by one to examine the gross lesions of the proximal tibia and femoral head in the leg bones. The lesions were scored according to their severity level following the BCO lameness lesion classification presented in Figure 3 [31,32].

### 2.3. Statistical Analyses

The identified lesions in the proximal femur and tibia from necropsied ducks were calculated and are reported as simple frequency statistics. A one-way analysis of variance (One-way ANOVA) was used to analyze the live body weight data using JMP Pro 18 (SAS Institute, Cary, NC, USA). Statistical significance was determined at *p* < 0.05.

## 3. Results

### 3.1. Live Body Weight

In total, 150 ducks aged 35 days (5 weeks) from three commercial farms demonstrated a total average live body weight (BW) of 1.42 ± 0.03 kg, while 50 ducks aged 37 days presented an average BW of 1.45 ± 0.04 kg (*p* = 0.2). The distribution of the average body weight of each farm and the percentage of females and males are presented in Table 1.

### 3.2. Lameness Lesions

**Figure 4:**
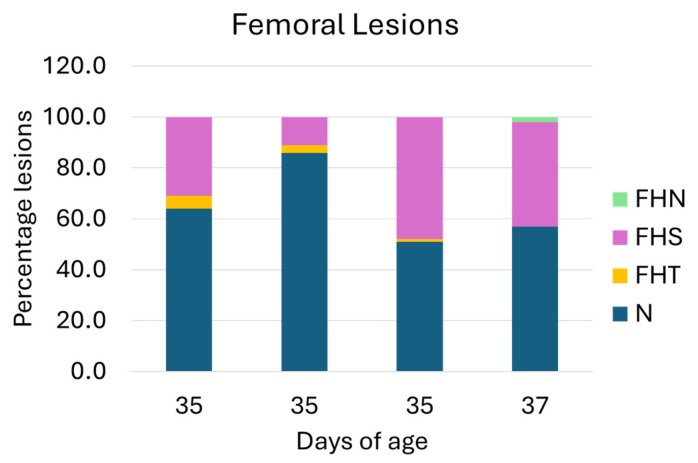
Percentage of femoral lesion categories. N = normal femoral head; FHS = proximal femoral head separation; FHT = proximal femoral head transitional degeneration; and FHN = proximal femoral head necrosis. Of the four flocks, N and FHS demonstrated the highest and the second-highest lesion categories.

**Figure 5:**
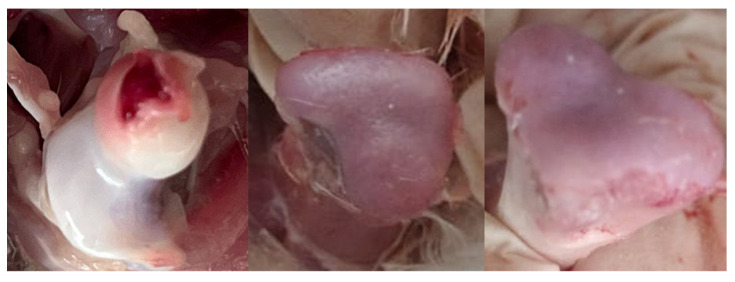
Pictures of femoral lesions found in ducks. Normal femur (**left**), femoral head separation (FHS) (**middle**), and femoral head transitional degeneration (FHT) (**right**).

The rate of lameness lesions of the legs was examined by necropsying the femoral head and proximal tibia. The necropsy on the femoral head of the leg bone showed that the lesions of N, FHS, FHT, and FHN of the birds aged 35 days were 67%, 30%, 3%, and 0%, while the lesions of the birds aged 37 days were 57%, 41%, 0%, and 2%, respectively. The distribution of lesion categories in ducks from four farms is presented in Figure 4. In total, 62% of the flocks possessed no clinical lesions (healthy), and approximately 39% showed subclinical lesions. The total distribution of FHS, FHT, and FHN lesions was 36%, 2%, and 1%. The typical femoral lesions found are presented in Figure 5.

**Figure 6:**
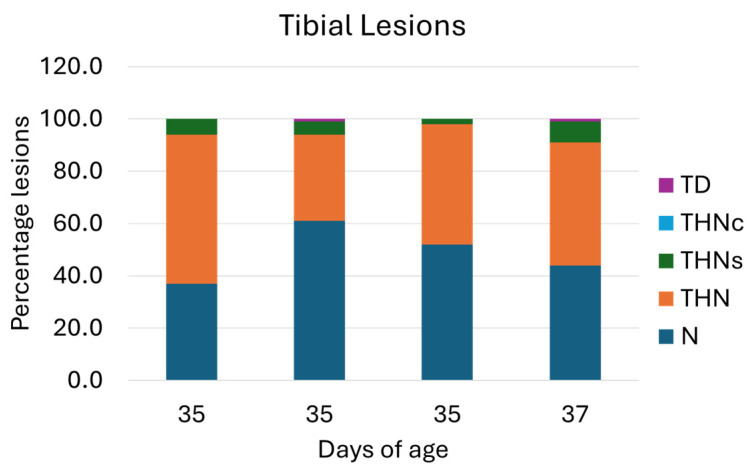
Percentage of tibial lesion categories. N = Normal Tibial Head; THN = Proximal Tibial Head Necrosis; THNc = Proximal Tibial Head Necrosis Caseous; THNs = Proximal Tibial Head Necrosis Severe; TD = Tibial Dyschondroplasia (another type of leg disorder). Of the four flocks, the rates of N and THN lesion categories seemed approximately equal.

**Figure 7:**
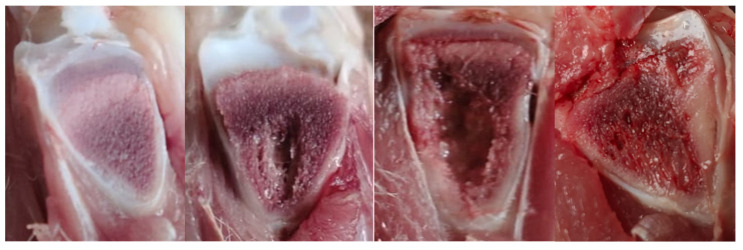
Pictures of tibial lesions found in ducks: from left to right, normal tibia, tibial head necrosis (THN), tibial head necrosis severe (THNS), and tibial dyschondroplasia (TD).

Furthermore, the distribution of tibial lesion categories in ducks from four farms is presented in Figure 6. The necropsy on the proximal tibia of the leg shows that 53% of the flocks presented healthy tibiae (N). Tibial lesions presenting THN, THNS, and TD severity levels (Figure 7) were 42%, 5%, and 1%, respectively. 

Generally, Figure 4 and Figure 6 demonstrate a parallel trend of the femoral and tibial lesions, where fewer lesions are observed in both femur and tibia in the second flocks. If the average of the tibial and femoral lesions is combined, 44% of the total surveyed duck population showed subclinical bone lesions. Of this 44%, approximately 2% (4 out of 200 ducks) showed signs of clinical lameness, particularly limping gait.

## 4. Discussion

The live body weight and lameness lesions in ducks were analyzed in this study. The average live BW of 35- and 37-day-old MP ducks grown by local farms in Mojokerto was not significantly different (1.42 ± 0.03 kg and 1.45 ± 0.04 kg, *p* = 0.2, respectively). This data is predicted to be approximately equal to the average body weight of female Mojosari ducks at slaughtering age (1.58 ± 0.09 kg), yet higher than the average body weight of female Alabio ducks (1.25 ± 0.12 kg) [15]. In addition to the breeder strain, feed/water intake, caging and flooring types, and growout management are several factors determining the performance and BW of ducks [18]. As briefly mentioned, the four commercial farms in this study used feed of a mixture of rice mill byproducts and Hi PROFIT 144 concentrate (PT. INARONEN POKPHAND Indonesia) at a 1:5 ratio, which aims to strike a balance between lower feed costs and marketable BW yield. Moreover, the ducks were also reared under a semi-intensive rearing system at a density of 1.51 m^2^/bird. In this model, the housing environment is simply intended as a shelter for the animals, limiting their access to outdoor areas and protecting them from predators and extreme weather, without extensive control over the temperature and humidity. The ducks are allowed to roam freely within the confines of the house during daylight hours with ad libitum access to feed and drinking water but are otherwise detached from natural behaviors—such as sunbathing, swimming, and foraging. Compared to intensive housing, where environmental conditions and feed intake are intensively controlled, ducks reared in extensive or semi-intensive rearing systems tend to have a smaller BW gain [33,34]. Taken together, these factors suggest that the smaller size of MP ducks produced in Mojokerto compared to Peking ducks in China is likely a result of multiple variables, including the breeder strain, formulated feed, rearing system, and stocking density.

As reported, approximately 2% (4 out of 200 ducks) of the surveyed duck population showed moderate clinical lameness signs, particularly a limping gait, and no immobilized birds were found. While somewhat limited in survey size, regular lameness incidence in duck flocks seems to be lower than that of broiler chickens. For instance, 19% of the birds in commercial broiler farms in Europe exhibited moderate-to-severe lameness symptoms [35], and 20 commercial broiler farms in Australia reported a 28% frequency of subclinical BCO lesions in necropsied birds [36]. As the commonly accepted postulation of BCO lameness pathogenesis involves the increased susceptibility of broiler leg joints and bones to mechanical injuries from the animal’s rapid weight gain, which allows for subsequent bacterial infection to take place [26,32], the smaller size and lower body weight in ducks potentially decrease the risk of BCO lameness incidence. In addition, ducks are reported to have superior immunity against diseases compared to other poultry species [12]. Preening of the skin involving oiling and nibbling is one of the exceptional innate immune responses of the duck, which is absent in other poultry species. While preening, the duck spread oil on the feathers, forming a natural repellent to some bacteria and fungi [37,38].

In this study, 44% of the necropsied flocks demonstrated FHS (36%) and THN (42%) as the most predominant lesions in the femoral head and proximal tibia, respectively. In FHS (femoral head separation, or epiphysiolysis), the articular cartilage is often damaged to various degrees, completely destroyed, or retained in the acetabulum and thus entirely separated from the proximal physis, leading to various observable states of the latter—ranging from undamaged to necrosis. THN (tibial head necrosis), on the other hand, represents mild necrotic voids in the metaphyseal zone of the tibia, which have otherwise not yet encroached upon the growth plate [39]. Together, the predominant frequency of FHS and THN represents a mild-to-moderate BCO lameness state. While birds possessing subclinical FHS and THN lesions may look seemingly healthy with no signs of clinical lameness [27], they may pose an increased threat to consumer safety from improper handling of duck meat during the slaughtering process, transportation, and cooking preparation—especially in the sale of whole birds, which bypasses further fabrication at processing plants and potential elimination of defective carcass parts, such as the leg. Furthermore, birds possessing FHS and THN lesions can develop into a severe BCO lameness state within 24–28 h if there is a sudden bacterial outbreak on the farms [27]. Interestingly, the lesions of FHN and THNS, representing a severe state of BCO lameness, including paralyzed or immobilized birds, were only recorded by 1% and 5%, respectively. While not directly comparable, this is generally considered a non-significant trend in terms of broiler lameness evaluation. Regardless, it is clear that much more preliminary research and investigation of BCO lameness in ducks need to take place in order to establish common baselines in evaluated parameters—including average industrial lameness incidence rate and etiological agents responsible for pathogenic infection—from which effective research induction models and effective preventive measures can be developed and investigated, much like the rapid pace at which the current field of BCO lameness research has developed in recent years [31,40,41].

## 5. Conclusions

The lameness survey in ducks presented 2% of clinically lame birds out of 44% of the surveyed population developing BCO lesions (subclinically lame). This incidence rate is lower than the lameness rate in broilers. Of the 44% BCO lesions found, FHS (36%) and THN (42%) were the most frequent lesions recorded in the femoral head and proximal tibia, respectively, representing a mild-to-moderate lameness state. The lameness survey conducted here is—to our knowledge—the first in the field in this geographical region. As such, this data may be valuable in the development of measures to increase performance, immunity, and structural bone of ducks. Lastly, further study on the identification of bacterial causative agents of BCO lameness in ducks is necessary to develop measures to control BCO lameness in the species.

## Figures and Tables

**Table 1:** The average body weights of the necropsied birds from each farm.

Age (Days)	#Birds	%Female/%Male	Average BW (kg)	SD
35	50	0.52/0.48	1.45 ^a^	0.04
35	50	0.36/0.64	1.42 ^a^	0.04
35	50	0.64/0.36	1.39 ^a^	0.06
37	50	0.26/0.74	1.45 ^a^	0.04

Note: BW = live body weight; SD = standard deviation; ^a^ represents non-statistical differences.

## Data Availability

The dataset used and/or analyzed in the study is available from the corresponding author (Adnan Alrubaye) upon request.
